# Catheter ablation in Asian patients with atrial fibrillation and hypertrophic cardiomyopathy: electrophysiological characteristics of recurrence and long-term clinical outcomes

**DOI:** 10.3389/fcvm.2023.1135230

**Published:** 2023-05-12

**Authors:** Chih-Hsien Lin, Chin-Yu Lin, Fa-Po Chung, Yenn-Jiang Lin, Shih-Lin Chang, Li-Wei Lo, Yu-Feng Hu, Tze-Fan Chao, Jo-Nan Liao, Ting-Yung Chang, Ta-Chuan Tuan, Ling Kuo, Cheng-I Wu, Chih-Min Liu, Shin-Huei Liu, Guan-Yi Li, Ming-Jen Kuo, Chi-Jen Weng, Shih-Ann Chen

**Affiliations:** ^1^ Division of Cardiology, Department of Internal Medicine, Chi-Mei Medical Center, Tainan, Taiwan; ^2^ Heart Rhythm Center, Taipei Veterans General Hospital, Taipei, Taiwan; ^3^ Department of Medicine, National Yang Ming Chiao Tung University, Taipei, Taiwan; ^4^ Cardiovascular Center, Taichung Veterans General Hospital, Taichung, Taiwan; ^5^ Vice President Office, National Chung Hsing University, Taichung, Taiwan

**Keywords:** atrial fibrillation, hypertrophic cardiomyopathy, catheter ablation, recurrence, long-term outcomes

## Abstract

**Background:**

Catheter ablation (CA) is a treatment strategy for atrial fibrillation (AF) in patients with hypertrophic cardiomyopathy (HCM). We investigated the electrophysiological characteristics of recurrence in a tertiary referral center and compared long-term clinical outcomes after CA therapy with patients who did not undergo CA.

**Methods:**

Patients with HCM and AF who underwent CA (group 1, *n* = 60) or pharmacological treatment (group 2, *n* = 298) between 2006 and 2021 were enrolled in this study. The baseline characteristics and electrophysiological characteristics of group 1 patients were examined to elucidate the reason for the recurrence of AF after CA therapy. The clinical results of the patients in Group 1 and Group 2 were compared using a propensity score (PS)-matched method.

**Results:**

The most common cause of recurrence was pulmonary vein reconnection (86.5%), followed by non-pulmonary vein triggers (40.5%), cavotricuspid isthmus flutter (29.7%), and atypical flutter (24.3%). Thyroid disease (HR, 14.713; *P *< 0.01), diabetes (HR, 3.074; *P *= 0.03), and non-paroxysmal AF (HR, 4.012; *P *= 0.01); these factors independently predicted recurrence. After the first recurrence, patients who underwent repeat CA showed a better arrhythmia-free state (74.1%) than those who underwent drug escalation therapy (29.4%, *P *< 0.01). After matching, PS-group 1 patients showed significantly better outcomes in all-cause mortality, heart failure hospitalization, and left atrial reverse remodeling than PS-group 2 patients.

**Conclusions:**

Patients who underwent CA showed better clinical outcomes than those who underwent drug therapy. The main predictors of recurrence were thyroid disease, diabetes, and non-paroxysmal AF.

## Introduction

Atrial fibrillation (AF) is the most common sustained arrhythmia in patients with hypertrophic cardiomyopathy (HCM), which appears to be 4- to 6-fold in patients with HCM more common than in the general population (1). The prevalence of AF was approximately 22% in a previous cohort of HCM (2). Diastolic dysfunction, mitral regurgitation, and obstruction of the left ventricular (LV) outflow tract in patients with HCM could increase the left atrial afterload and vulnerability to the occurrence of AF, further promoting LA dilatation and electric remodeling, resulting in a vicious circle (2). Atrial fibrillation is a significant contributor to morbidity and mortality in patients with HCM as it increases the risk of ischemic stroke, worsens symptoms of heart failure (HF), and causes functional disability and death (3–5). Despite the relatively high incidence of AF in patients with HCM and the association of morbidity with this arrhythmia, only a few studies have systematically addressed the clinical implications of AF ablation in patients with HCM.

Catheter ablation (CA) is a well-accepted therapeutic strategy for treating AF. However, the success rates of CA in patients with HCM are lower than in patients without HCM (6, 7), and continued procedures after initial treatment are often necessary to restore long-term sinus rhythm (8). One previous report with a propensity score (PS)-matched suggested that CA protected the renal function (9); however, data on long-term clinical outcomes of patients with HCM undergoing radiofrequency catheter ablation (RFCA) are not well-established. The present study aimed to investigate the electrophysiological characteristics of recurrent AF and the impact of RFCA on long-term outcomes of patients with HCM compared with patients undergoing pharmacological treatment alone.

## Methods

### Study population

The present retrospective cohort study included consecutive patients with documented HCM and AF who underwent CA for drug-refractory AF (group 1) and those who did not receive CA (group 2) between June 2006 and October 2021 at Taipei Veterans General Hospital. The clinical tachycardia observed was only AF in our study cohort. The baseline characteristics, echocardiographic parameters and electrophysiological findings of the patients were assessed. The diagnosis of HCM was based on two-dimensional echocardiographic evidence of a non-dilated hypertrophied left ventricle (maximum wall thickness ≥ 15 mm) in the absence of any other cardiac or systemic disease capable of inducing the magnitude of evident hypertrophy. AF was diagnosed on the basis of electrocardiographic recordings or Holter monitoring in the hospital. AF was defined as paroxysmal if the episodes ended spontaneously or with intervention, within 7 days after the onset. All patients who had a history of thyroid disease were now under euthyroid status. The Institutional Ethics Review Board of Taipei Veterans General Hospital approved this study (IRB No. 2021-11-015BC). Given the retrospective nature of the study, the institutional review board waived the requirement for informed consent.

### Exclusion criteria

Patients who had undergone a maze procedure previously, had a history of valvular AF, alcohol septal ablation, had undergone surgical myectomy, or were unable to visit our hospital or receive a telephone interview after enrollment were excluded from this study.

### The ablation procedure in the group 1 patient

The details of the protocol have been described in our previous studies (10, 11). All antiarrhythmic drugs, except amiodarone, were discontinued for at least five half-lives before the procedure. A 7F decapolar catheter with a distance of 2 mm between electrodes and a spacing of 5 mm between each electrode pair was inserted into the coronary sinus through the right internal jugular vein. Transseptal atrial puncture was performed using fluoroscopic landmarks and an 8.5F SL-0 sheath (St. Jude Medical, Inc.). Patients with paroxysmal AF underwent pulmonary vein isolation (PVI) and non-pulmonary vein (PV) trigger ablation. The CA procedure was performed as described in our previous studies (10). The cavotricuspid isthmus (CTI) line was performed during the procedure in patients with a history of isthmus-dependent atrial flutter or induced isthmus-dependent atrial flutter. AF was induced in patients with paroxysmal AF at the end of the ablation procedure. If LA flutter sustained for more than 1 min, the reentry circuit of LA flutter was identified by isochrone mapping, entrainment maneuvers, and postpacing interval analysis, followed by linear ablation to eliminate atrial flutter. If the AF was still inducible, cardioversion was performed if the AF did not end spontaneously. The endpoints of the procedure were the entrance/exit block of the pulmonary veins and the elimination of non-PV triggers (11).

PVI was performed as previously described in patients with non-paroxysmal AF. If AF recurred after the first step, additional linear ablation or complex atrial fractionated electrographically guided substrate ablation was performed at the operator's discretion. If AF recurred after ablation, sinus rhythm was restored through electric cardioversion. Non-PV trigger ablation is performed routinely in our laboratory. If AF became organized during ablation, electroanatomic mapping and radiofrequency ablation were performed to stop organized tachycardia (11). In patients with non-paroxysmal AF, if AF recurred or was inducible after the ablation procedure, an additional complex fractionated atrial electrogram (CFAE) ablation was performed before 2015.

In patients undergoing repeat procedures, the CA strategy was similar to the methods described in the index procedure. Furthermore, the cause of the recurrence was investigated.

### Follow-up and definition of arrhythmia recurrence

After discharge, patients were followed-up (two weeks after CA, then every 1 to 3 months) in our cardiology clinic or by referring physicians. Routine electrocardiograms (ECGs) were obtained at each outpatient visit, and a 24-h Holter electrocardiogram was performed at 3, 6, and 12 months. When patients experienced symptoms suggestive of tachycardia after ablation, 24-h Holter monitoring or cardiac event recording was performed to define the cause of clinical symptoms. Recurrence was defined as any episode of documented AF or atrial tachycardia > 30s after the initial 3 months of the blank period. A telephone interview was conducted with all patients in May 2022.

### Propensity score matching

To minimize the impact of confounding factors on clinical characteristics, we employed propensity analysis and matching techniques. We matched pairs one-to-one (Group 1 vs. Group 2) with identical propensity scores and a 0.01 caliper width. The suitability was assessed by estimating the standardized differences between the two groups for age, sex, hypertension, HF, type of AF and left atrial diameter (LAD) (12).

### Outcome assessment

Patients received scheduled follow-ups every 1 to 3 months, depending on their clinical course. The follow-up data of all participants was retrieved from Taipei Veterans General Hospital Database. The primary endpoint was the all-cause mortality rate. Secondary endpoints were cardiovascular (CV) death, acute myocardial infarction (AMI), hospitalization for HF, cerebrovascular accident (CVA) and changes in LAD and left ventricular ejection fraction (LVEF) on echocardiography. CV death included those resulting from AMI, sudden cardiac death, HF, stroke, and other CV causes. CVA included stroke and transient ischemic attack. Primary and secondary endpoints were investigated in detail based on medical records from the Taipei Veterans General Hospital, the Ministry of Health and Welfare and telephone interviews. The overall follow-up period extended upto May 2022.

### Statistical analysis

Descriptive statistics are reported as mean values and standard deviation values for continuous variables, and percentages for categorical variables. Comparisons between groups were made using the unpaired Student's t-test for continuous data and the chi-square test for categorical variables. A multivariate Cox regression model was used to identify predictors of recurrence. The variables selected for the multivariate analysis were parameters with *P *< 0.1 in the univariate model. Long-term survival curves were plotted using the Kaplan-Meier method and statistical significance was examined using the log-rank test. Relative change was defined as the difference between follow-up and baseline values divided by the baseline value. All tests were two-sided and statistical significance was set at *P *< 0.05.

## Results

### Study population

The study enrolled 358 patients with HCM and AF. Sixty patients underwent CA (group 1), and 298 did not undergo CA (group 2). The baseline characteristics of the two study groups are summarized in Supplementary Table S1. After applying propensity score matching to balance the characteristics between patient groups, 49 patients treated with CA (PS-group 1) and 49 patients without CA (PS-group 2) were compared. The baseline characteristics of the two groups were balanced (Table 1).

**Table 1:** Baseline characteristics of 49 patients in PS-group 1 and 49 patients in PS-group 2 after propensity score matching.

	PS-group 1 (*n* = 49)	PS-group 2 (*n* = 49)	*P*-value
Age (Mean ± SD)	58.8 ± 9.9	58.5 ± 13.1	0.91
Male (*n*, %)	40 (81.6%)	42 (85.7%)	0.59
BMI (Mean ± SD)	27.0 ± 3.8	25.6 ± 4.3	0.12
CHA_2_DS_2_-VASc (Mean ± SD)	1.57 ± 1.25	1.39 ± 1.29	0.48
Hypertension (*n*, %)	17 (34.7%)	22 (44.9%)	0.30
Hyperlipidemia (*n*, %)	15 (30.6%)	13 (26.5%)	0.66
Diabetes (*n*, %)	12 (24.5%)	12 (24.5%)	1.00
Coronary artery disease (*n*, %)	11 (22.5%)	9 (18.4%)	0.62
Congestive heart failure (*n*, %)	7 (14.3%)	6 (12.2%)	0.77
Vascular disease (*n*, %)	1 (2.0%)	1 (2.0%)	1.00
Cerebrovascular disease (*n*, %)	4 (8.2%)	3 (6.1%)	1.00
Obstructive sleep apnea (*n*, %)	3 (6.1%)	1 (2.0%)	0.62
Thyroid disease (*n*, %)	5 (10.2%)	3 (6.1%)	0.72
Type of AF			0.69
Paroxysmal	28 (57.1%)	26 (53.1%)	
Non-paroxysmal	21 (42.9%)	23 (46.9%)	
LAD (mm) (Mean ± SD)	44.8 ± 7.0	45.5 ± 7.9	0.65
LVEF			1.00
>50%	38 (77.6%)	37 (75.5%)	
40–50%	8 (16.3%)	9 (18.4%)	
<40%	3 (6.1%)	3 (6.1%)	

AF, atrial fibrillation; BMI, body mass index; LAD, left atrial diameter; LVEF, left ventricular ejection fraction; PS, propensity score matching; SD, standard deviation.

### Procedural details of the index catheter ablation

**Figure 1:**
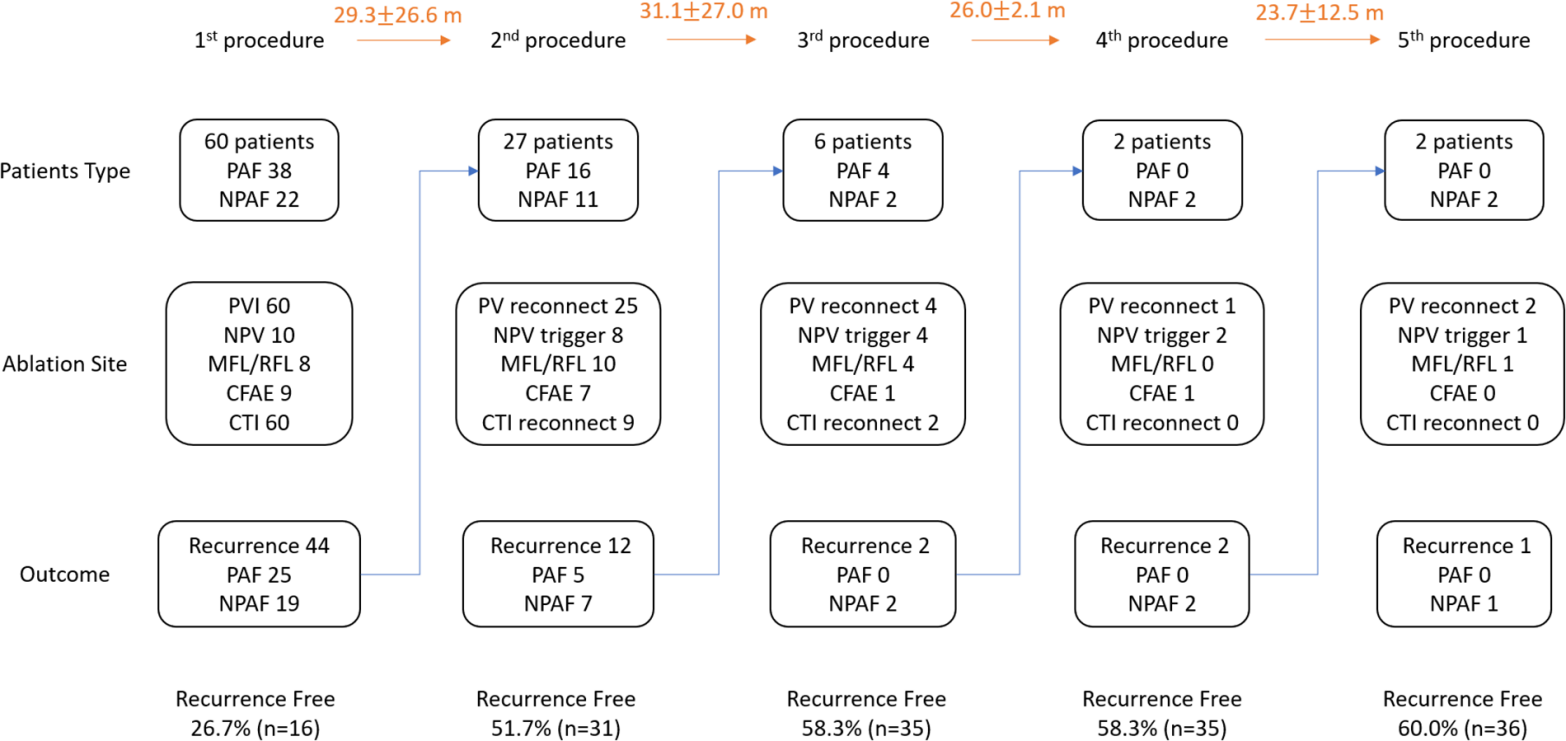
Ablation strategy and outcomes. The figure demonstrated the ablation strategies and long-term outcomes. CFAE, complex fractionated atrial electrogram; CTI, cavotricuspid isthmus; MFL, mitral flutter; NPAF, non-paroxysmal atrial fibrillation; NPV, non-pulmonary vein; PAF, paroxysmal atrial fibrillation; PV, pulmonary vein; PVI, pulmonary vein isolation; RFL, roof flutter.

There were 38 patients (63.3%) with paroxysmal AF in Group 1. PVI and CTI ablations were performed in all patients in the index procedure (Figure 1). Among the 22 patients with non-paroxysmal AF, seven underwent CFAE ablation and five received linear ablation. Non-PV triggers were identified in three patients with paroxysmal AF from the LA posterior wall (two) and superior vena cava (SVC) (one) and in seven patients with non-paroxysmal AF from the LA posterior wall (three), LA septum (two), vein of Marshall (one) and SVC (one).

### Recurrence patterns and causes of recurrence

After the first procedure, 44 of 60 patients (73.3%) experienced AF recurrence and 27 (61.3%, 27/44) underwent a second ablation procedure. Twelve patients experienced a recurrence of AF after the second procedure, and six (50.0%, 6/12) underwent the third procedure. Two patients had AF recurrence after the third procedure and two (100%) underwent the fourth and fifth procedures. In the 37 repeat procedures, the most common cause of recurrence was PV reconnection (86.5%, 32/37), followed by non-PV triggers (40.5%, 15/37), CTI reconnection (29.7%, 11/37), and mitral or roof line reconnection (24.3%, 9/37). New atypical flutter was observed in 16.2% of the patients (6/37, two mitral flutters and four roof flutters).

### Follow-up after catheter ablation

**Figure 2:**
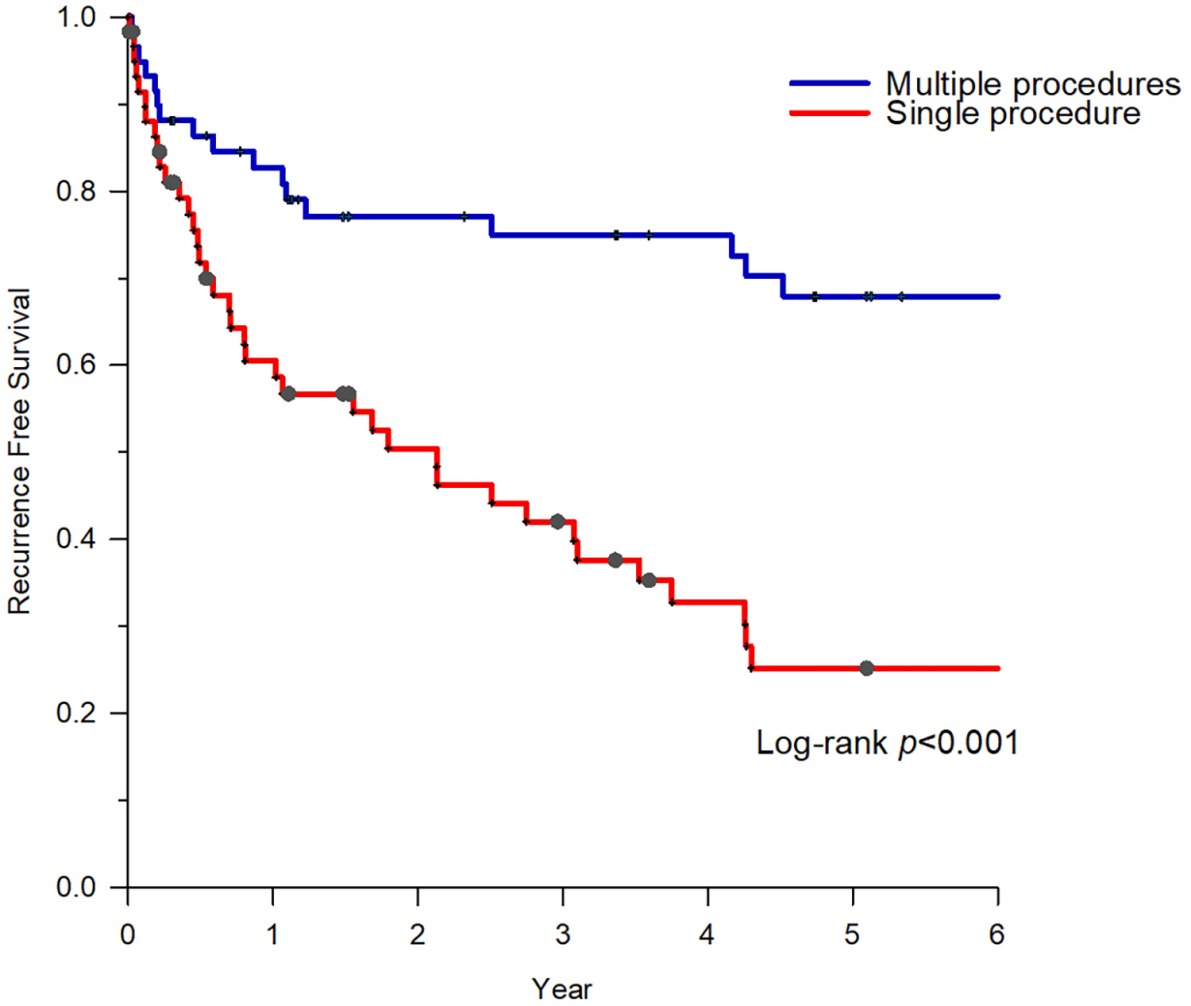
Survival of freedom from atrial arrhythmias of group 1. After an average of 1.6 ± 0.9 times of ablation, patients with HCM who underwent repeat CAs demonstrated significantly better atrial arrhythmia-free status. CA, catheter ablation; HCM, hypertrophic cardiomyopathy.

The index procedure was recurrence-free in 16 (26.7%) patients. The freedom from recurrence reached 60% (36/60) after the final procedure. Following the index procedure, AF recurrence was observed in 44 patients (73.3%), 17 of whom (38.6%) underwent drug escalation therapy instead of CA. Patients who underwent repeated CA demonstrated significantly improved atrial arrhythmia-free status (74.1%, 20/27) than those who underwent drug escalating therapy (29.4%, 5/17, *P *< 0.01). After a mean follow-up of 7.1 ± 4.2 years, with an average of 1.6 ± 0.9 times ablation, 41 of 60 patients (68.3%) in group 1 were completely free from atrial arrhythmias (Figure 2).

### Predictors of recurrence after catheter ablation

Multivariate analysis demonstrated that diabetes mellitus (DM) (hazard ratio [HR]: 3.074, 95% confidence interval [CI]: 1.089–8.678, *P *= 0.03), thyroid disease (HR: 14.713, 95% CI: 3.972–54.497, *P *< 0.01), and non-paroxysmal AF (HR: 4.012, 95% CI: 1.476–10.905, *P *< 0.01) were independent predictors of AF recurrence after ablation (Table 2).

**Table 2:** Predictors of recurrence after catheter ablation. .

	Univariable		Multivariable	
Parameters	HR (95% CI)	*p*-value	HR (95% CI)	*p*-value
Age	1.023 (0.975–1.072)	0.36		
Male	1.333 (0.379–4.684)	0.65		
Hypertension	0.659 (0.250–1.739)	0.40		
Hyperlipidemia	1.279 (0.498–3.284)	0.61		
Diabetes mellitus	2.805 (1.113–7.072)	0.03	3.074 (1.089–8.678)	0.03
Coronary artery disease	2.631 (1.047–6.613)	0.04	1.986 (0.733–5.379)	0.18
Congestive heart failure	1.280 (0.293–5.589)	0.74		
Thyroid disease	7.376 (2.304–23.611)	<0.01	14.713 (3.972–54.497)	<0.01
Cerebrovascular accident	1.495 (0.343–6.509)	0.59		
Obstructive sleep apnea	3.574 (0.797–16.023)	0.10	3.926 (0.752–20.496)	0.11
CHA_2_D_2_-VASc	1.245 (0.873–1.777)	0.23		
Non-paroxysmal AF	3.392 (1.311–8.780)	0.01	4.012 (1.476–10.905)	<0.01
LAD	1.029 (0.968–1.094)	0.36		
LVEF	0.987 (0.939–1.038)	0.60		
IVSd	0.956 (0.849–1.076)	0.45		
LVPWd	1.059 (0.954–1.175)	0.28		

AF, atrial fibrillation; CI, confidence interval; HR, hazard ratio; IVSd, interventricular septal width in diastole; LAD, left atrial diameter; LVEF, left ventricular ejection fraction; LVPWd, left ventricle posterior wall thickness in diastole.

### Long-term clinical outcomes

During the follow-up period of 7.1 ± 4.3 years, none of the patients in group 1 experienced mortality, CVA, AMI, or HF hospitalization. On the contrary, all-cause mortality occurred in 76 of 298 patients (25.5%) in Group 2. CV death occurred in 19 of 298 patients (6.4%), while CVA occurred in 36 of 298 patients (12.1%). Acute myocardial infarction and hospitalization with HF occurred in five (1.7%) and 99 (33.2%) patients, respectively. All-cause mortality, CVA and hospitalization for HF were significantly higher in group 2 than in group 1 during the follow-up period (Supplementary Figure S1).

**Figure 3:**
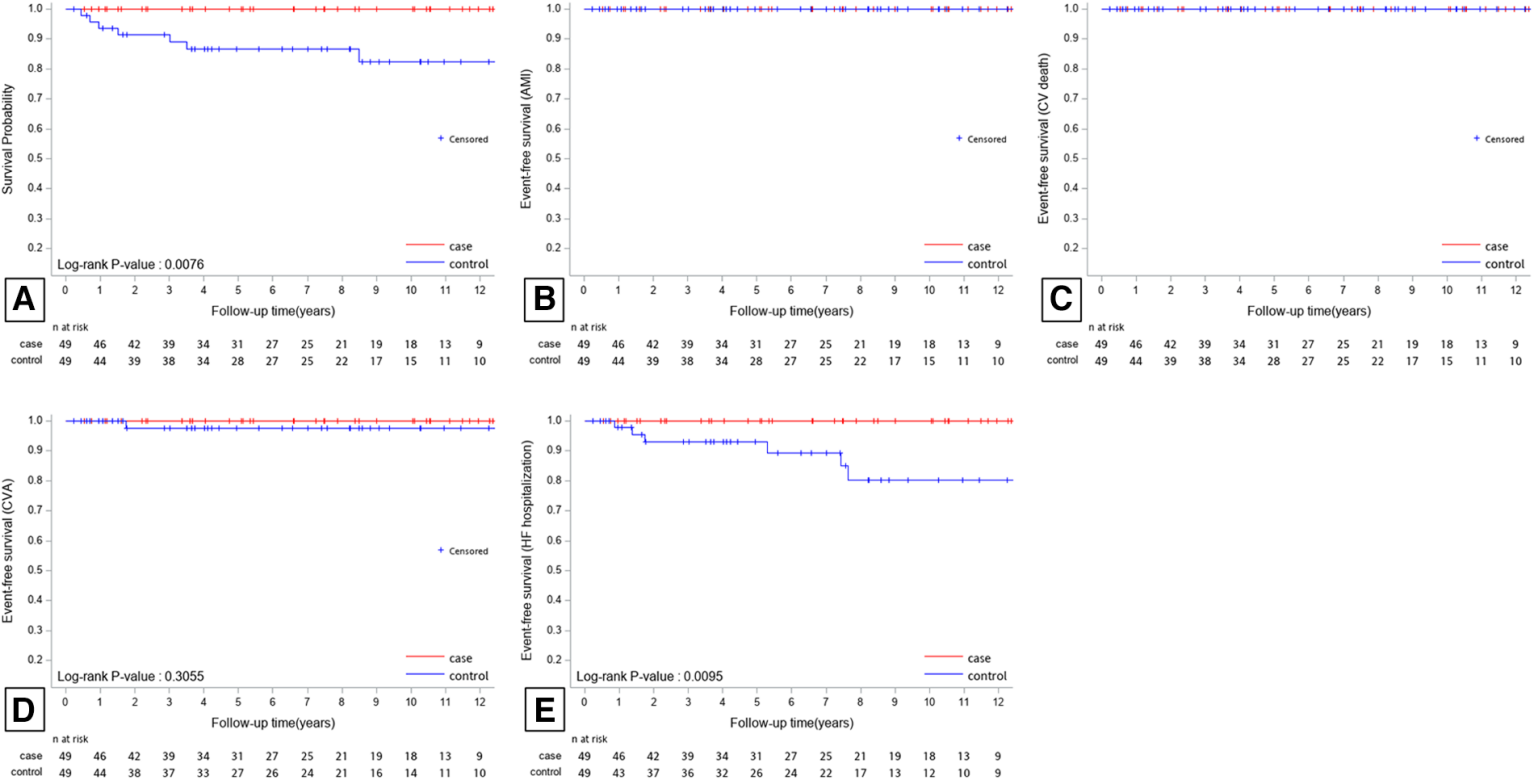
Kaplan-Meier survival plots for all-cause mortality (**A**), AMI (**B**), CV death (**C**), CVA (**D**), and HF hospitalization (**E**) of the PS-matched patients. All-cause mortality and HF hospitalization were significantly higher in PS-group 2 than in PS-group 1 during a mean follow-up period of 7.2 ± 4.2 years. AMI, acute myocardial infarction; CV, cardiovascular; CVA, cerebrovascular accident; HF, heart failure.

After applying propensity score matching, 49 patients treated with CA (PS-group 1) and 49 patients without CA (PS-group 2) were compared. No AMI or CV death events occurred in PS-group 1 during follow-up. All-cause mortality occurred in seven patients (14.3%) in PS-group 2. Hospitalization for CVA and HF was observed in one patient (2.0%) and six patients (12.2%) in PS-group 2, respectively. The Kaplan-Meier curves for all-cause mortality, CVA, and hospitalization for HF in PS-matched patients are presented in Figure 3. All-cause mortality and hospitalization for HF were significantly higher in PS group 2 than in PS group 1 during a mean follow-up period of 7.2 ± 4.2 years.

### Comparison of echocardiogram parameters

We compared LAD and LVEF of PS-matched patients. Forty-six patients in PS-group 1 and 40 in PS-group 2 underwent an echocardiogram examination at the end of follow-up (Table 3). The LAD of PS-group 1 decreased significantly at the end of follow-up compared to the baseline LAD (*P *= 0.04). For PS-group 2, the LAD increased significantly at the end of follow-up compared to the baseline LAD (*P *< 0.01). The relative changes in LAD between the two groups during the follow-up period were significant (PS-group 1 vs. PS-group 2: −2.7 ± 12.9% vs. 6.4 ± 14.3%, *P *< 0.01). There was no significant change in LVEF at the end of follow-up in PS-group 1 (*P *= 0.52) and PS-group 2 (*P *= 0.43). The relative changes in LVEF between these two groups at the end of follow-up was also not significantly different (PS-group 1 vs. PS-group 2: 5.6 ± 28.4% vs. −0.84 ± 13.5%, *P *= 0.20).

**Table 3:** Comparison of baseline and follow-up echocardiogram parameters of the propensity-matched patients.

Parameters	PS-group 1	PS-group 2	*P*-value
	(*n* = 46)	(*n* = 40)	
**LAD (mm)**			
Baseline	45.0 ± 7.2	46.6 ± 7.2	0.34
End of follow-up	43.2 ± 6.3^a^	49.4 ± 9.5^a^	<0.01
Relative change (%)	−2.7 ± 12.9	6.4 ± 14.3	<0.01
**LVEF (%)**			
Baseline	56.4 ± 10.2	55.4 ± 9.3	0.65
End of follow-up	57.5 ± 8.3	54.5 ± 8.9	0.12
Relative change (%)	5.6 ± 28.4	−0.84 ± 13.5	0.20

^a^ significant (*p* < 0.05) when compared with baseline.

LAD, left atrial diameter; LVEF, left ventricular ejection fraction; PS, propensity score matching.

## Discussion

### Main finding

The present study is the first to compare CA of AF with pharmacological therapy in the HCM population using PS-matching, and it has several significant findings. First, complete recovery from AF could be achieved in patients with HCM after performing single or multiple RFCA for AF. Second, PV reconnection was the most common cause of recurrence. Thyroid disease, DM, and non-paroxysmal AF were independent predictors of recurrence. Third, patients with AF and HCM who received RFCA showed significantly better all-cause mortality and HF hospitalization outcomes than those who did not undergo CA. Fourth, LA dilatation in patients with AF and HCM could be significantly improved after CA therapy during follow-up compared with that in patients who only received pharmacological treatment.

### Recurrence in HCM patients with AF after RFCA

Castagno et al. conducted a retrospective cohort study that included 116 patients with HCM and AF who underwent RFCA. Over a median follow-up of six years, with an average of 1.6 procedures, 67 (61%) patients showed sinus rhythm (SR) (13). Dinshaw et al. retrospectively enrolled 65 patients with HCM who underwent AF ablation. After 1.9 ± 1.2 ablation procedures and a follow-up of 48.1 ± 32.5 months, no recurrence was observed in 60.0% of the patients (14). Similarly, Zheng et al. retrospectively evaluated the outcome of 120 patients with HCM after AF ablation; after a single procedure, 70 (58.3%) patients experienced AF recurrence. After repeat procedures, 82 (68.3%) of 120 patients recovered completely from AF/AT as per the last follow-up (15). Previous studies showed a long-term arrhythmia-free survival rate between 60% and 70%. Our results are comparable to previous studies. Santangeli et al. noted that non-PV triggers are responsible for late recurrences (16). In the present study, we further demonstrated that the most common recurrent pattern in AF ablation in HCM patients was PV reconnection, followed by non-PV triggers and LA flutter. A previous study reported that AF recurrence after the first isolation of the PV antrum is higher in patients with HCM (17). The cause of PV reconnection reported in our study may be the presence of thickened atrial muscle resulting from high LA pressure, atrial fibrosis and the nature of cardiomyopathy (17).

In patients with HCM with AF recurrence after the first CA, the benefits of repeat CA or drug escalation therapy have not been investigated. We showed that patients who underwent repeat CA had a significantly better atrial arrhythmia-free status than those who underwent drug escalation therapy (*P *< 0.01). Repeat CA can be an effective method for controlling rhythm in patients with HCM and recurrent AF.

### Catheter ablation vs. pharmacological therapy in HCM patients with AF

Higuchi et al. compared patients with HCM treated for AF with CA (*n* = 34) and those who did not undergo CA (*n* = 60). They found that the combined incidence of clinical events, including HCM-related death, hospitalization for HF or new-onset thromboembolic strokes, was significantly lower in the CA group than in the non-CA group (*P *= 0.03) during a mean follow-up of 5.8 years (18). Zheng et al. also compared the composite clinical event rates after ablation between HCM patients with (*n* = 120) and without CA (*n* = 32), which included all-cause mortality, unplanned hospitalization for HF, and new-onset thromboembolic stroke. The results showed that the composite rate of clinical events was lower in the CA group than in the non-CA group (*P *= 0.02) (15). In our study, we consecutively enrolled patients with HCM and AF from 2006 to 2021, and investigated long-term clinical events individually, rather than studying composite results. The results showed significantly good outcomes in terms of all-cause mortality and hospitalization due to HF. In patients with HCM, AF is usually poorly tolerated due to loss of atrial contraction, with worsening diastolic dysfunction. In addition to the impact on diastolic function, several reports have indicated that patients with HCM who develop AF have a higher risk of mortality, ischemic stroke, and exacerbation of HF than those without AF (3–5, 19, 20). The association between AF and mortality and morbidity suggests that AF should be carefully and skillfully managed in patients with HCM. Our results suggest that rhythm control by RFCA in patients with HCM and AF can reverse or slow the process to a disappointing prognosis.

### Change of LAD in HCM patients with AF

The occurrence of AF in patients with HCM is likely due to increased LA pressure and volume (4). Diastolic dysfunction and reduced LV compliance due to myocyte hypertrophy and disorganization play a key role in the enlargement of the left atrium (1). Dilated LA volume is correlated with the appearance of AF in patients with HCM, which implies the critical role of the vicious cycle between AF and LA volume in HCM. Therefore, an aggressive rhythm control strategy with CA can interrupt the vicious cycle and improve outcomes in patients with HCM and AF.

In the present study, we demonstrated an improvement in LA dilatation after AF ablation in patients with HCM compared with the non-CA patients (Table 3). This result may imply that LA enlargement is not only a precipitator of the development of AF, but also a secondary phenomenon of AF. RFCA rhythm control may slow the LA remodeling process and delay the comorbidity of AF and HCM. The study also did not show a significant change in LVEF at the end of follow-up in both PS-group 1 and PS-group 2. Common mechanisms of HF in patients with HCM include LVOT obstruction and diastolic LV dysfunction. The left ventricular ejection fraction is typically preserved in patients with HCM. It is reasonable that AF ablation did not affect LVEF. To our knowledge, this is the first study to investigate LAD and LVEF after RFCA in patients with HCM and AF. Due to the retrospective nature of this study, more studies are needed to clarify the relationship between AF ablation and LA remodeling in patients with HCM.

### Limitation

First, the number of subjects included in the present retrospective study was small, especially in the context of the prediction of the risk of recurrence of AF after ablation, and the primary endpoint of mortality. Second, the number of adverse outcomes in Group 1 was relatively small. The study cohort was retrospectively enrolled in this study. Selection bias is one of the limitations and may introduce the difference in the baseline characteristics and outcomes. We applied propensity score matching to attenuate selection bias. More randomized controlled studies are required to validate these results. Third, the diagnosis of HCM was based on clinical and echocardiographic findings. MRI and genetic testing were not performed routinely in all patients. Fourth, ECG or Holter monitoring was performed only during routine follow-up. Therefore, some patients with a low burden of asymptomatic paroxysmal AF may not have been identified. Fifth, the number of patients who underwent repeat CA in Group 1 was relatively small, which may limit the study findings on the mechanisms of recurrence.

## Conclusions

Although a single CA shows a high recurrence rate of AF, multiple CAs are effective for long-term control of AF. The main predictors of recurrence were thyroid disease, DM, and non-paroxysmal AF. PV reconnection is the most common cause of recurrence. Patients who underwent CA had better clinical outcomes, with lower mortality and hospitalization rates for HF. Reverse LA remodeling was observed in patients who underwent invasive electroanatomic mapping and CA. Additionally, repeat CA is better than drug escalation therapy for patients who experience AF recurrence after the first CA.

## Data Availability

The original contributions presented in the study are included in the article/**Supplementary Material**, further inquiries can be directed to the corresponding author/s.
