# Supplementary material for: Catheter ablation in Asian patients with atrial fibrillation and hypertrophic cardiomyopathy: electrophysiological characteristics of recurrence and long-term clinical outcomes
Source: Front Cardiovasc Med. 2023 May 12;10:1135230. doi: 10.3389/fcvm.2023.1135230 (PMC10213660; doi:10.3389/fcvm.2023.1135230)
Supplement: Supplementary file 1 [file Table2.docx]

**Supplemental Table 1.** Baseline characteristics of 60 patients in group 1 and 298 patients in group 2.

|  | Group 1 (n=60) | Group 2 (n=298) | *P*-value |
| --- | --- | --- | --- |
| Age (Mean±SD) | 57.5±10.3 | 74.0±12.8 | <0.01 |
| Male (n, %) | 51 (85.0%) | 223 (74.8%) | 0.09 |
| BMI (Mean±SD) | 26.8±3.6 | 24.8±4.9 | <0.01 |
| CHA_2_DS_2_-VASc  (Mean±SD) | 1.43±1.21 | 2.47±1.31 | <0.01 |
| Hypertension (n, %) | 22 (36.7%) | 127 (42.6%) | 0.39 |
| Hyperlipidemia (n, %) | 18 (30.0%) | 71 (23.8%) | 0.31 |
| Diabetes (n, %) | 13 (21.7%) | 69 (23.2%) | 0.80 |
| Coronary artery disease (n, %) | 12 (20.0%) | 77 (25.8%) | 0.34 |
| Congestive heart failure (n, %) | 7 (11.7%) | 96 (32.2%) | <0.01 |
| Vascular disease (n, %) | 1 (1.7%) | 8 (2.7%) | 1.00 |
| Cerebrovascular disease (n, %) | 4 (6.7%) | 19 (6.4%) | 1.00 |
| Obstructive sleep apnea (n, %) | 4 (6.7%) | 2 (0.7%) | <0.01 |
| Thyroid disease (n, %) | 6 (10.0%) | 12 (4.0%) | 0.10 |
| Type of AF |  |  | <0.01 |
| Paroxysmal | 38 (63.3%) | 127 (42.6%) |  |
| Non-paroxysmal | 22 (36.7%) | 171 (57.4%) |  |
| LAD (mm)  (Mean±SD) | 44.3±6.8 | 50.9±9.9 | <0.01 |
| LVEF |  |  | 0.55 |
| >50% | 49 (81.7%) | 228 (76.5%) |  |
| 40-50% | 8 (13.3%) | 43 (14.4%) |  |
| <40% | 3 (5.0%) | 27 (9.1%) |  |

AF, atrial fibrillation; BMI, body mass index; LAD, left atrial diameter; LVEF, left ventricular ejection fraction; SD, standard deviation.

**Supplemental Table 2.** Procedural data of 60 patients in group 1 patients.

|  | Group 1 (n=60) |
| --- | --- |
| Procedure time (min) | 186.0±41.2 |
| Radiation time (min) | 80.7±41.7 |
| Complications | 4 (6.7%) |
| Tamponade | 1 (1.7%) |
| Femoral pseudoaneurysm | 3 (5.0%) |


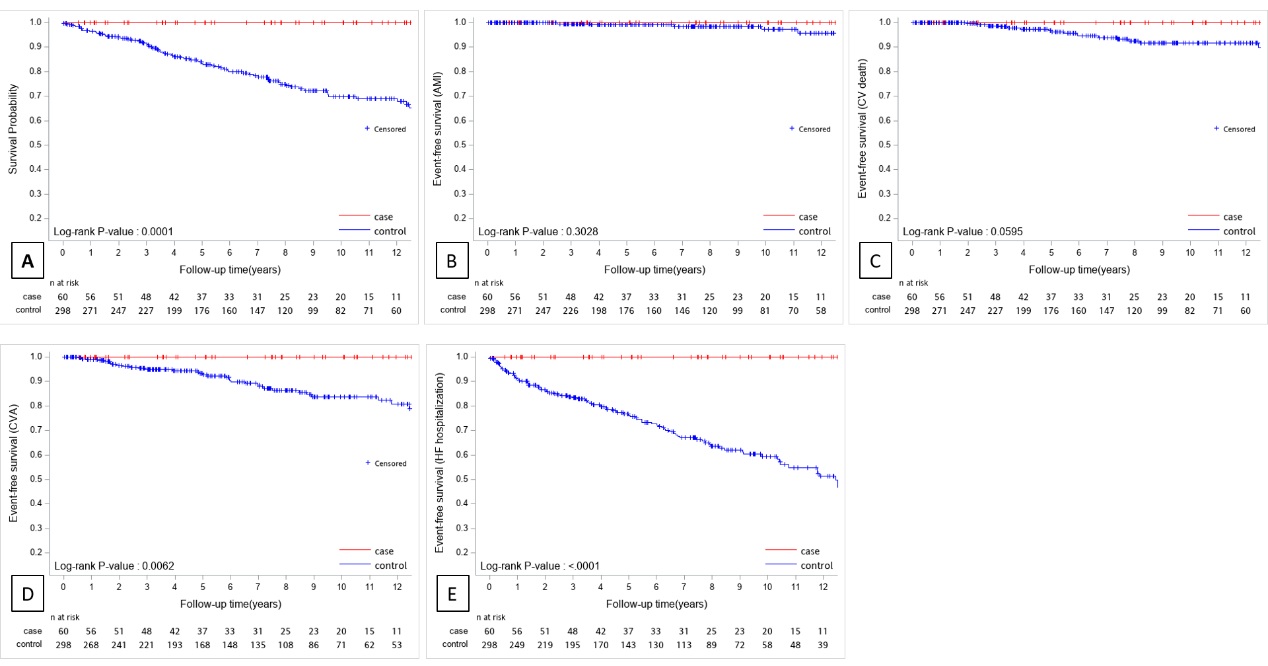


**Supplemental Figure 1.** Kaplan-Meier survival plots for all-cause mortality (A), AMI (B), CV death (C), CVA (D), and HF hospitalization (E) in group 1 (n=60) and group 2 (n=298).

All-cause mortality, CVA, and HF hospitalization were significantly higher in group 2 than in group 1 during the follow-up period of 7.1±4.3 years.

AMI, acute myocardial infarction; CV, cardiovascular; CVA, cerebrovascular accident; HF, heart failure.
